# The Coalition for Epidemic Preparedness Innovations (CEPI) and the Partnerships of Equitable Vaccine Access

**DOI:** 10.1017/jme.2023.85

**Published:** 2023

**Authors:** Sam Halabi, Lawrence O. Gostin, Kashish Aneja, Francesca Nardi, Katie Gottschalk, John Monahan

**Affiliations:** 1: O’NEILL INSTITUTE FOR NATIONAL AND GLOBAL HEALTH LAW, WASHINGTON, DC, USA; 2: COLORADO STATE UNIVERSITY, FORT COLLINS, CO, USA; 3: COLORADO SCHOOL OF PUBLIC HEALTH, AURORA, CO, USA; 4: GEORGETOWN UNIVERSITY, WASHINGTON, DC, USA

**Keywords:** Equitable Vaccine Access, Public Health Partnerships, Coalition for Epidemic Preparedness Innovations (CEPI), Pandemic Response, COVID-19 Vaccine

## Abstract

This article highlights and evaluates the role of CEPI and its contribution to global equitable access to COVID-19 vaccines through its established partnerships for vaccine development. The article adds to the understanding of how and when such partnerships can work for public health, especially under emergency citations.

## I. Introduction

Of the weaknesses in global health security exposed by the response to the COVID-19 pandemic, innovation, supply chains, and systems for equitable access were principal among them. Partnerships built by scientists across national borders and public- and private-sector institutions to advance vaccines targeting many diseases were transformed into “national” assets to be seized and used primarily and, at least preliminarily, exclusively for the benefit of national populations. The U.S., for example, deployed “Operation Warp Speed,” under which it used direct funding to take stakes in six vaccine candidates. By March 2021, these contractual funding and procurement agreements accounted for the purchase of over 1 billion doses by the U.S. government, all of which were dedicated to the U.S. population.^1^ French, German, and Indian governments followed by either taking stakes in vaccine manufacturers or issuing declarations aimed at their domestic producers.^2^

For their part, the large companies who held ownership over key inputs, especially the drug substances themselves, behaved consistently with what theories of shareholder-owned enterprise and capital market discipline would predict: they sought to maximize profits and return to their investors.^3^ The arguable exception was AstraZeneca, which made an early commitment to sell doses on a no-profit basis, due in significant part to the demands of its university partners and their supporters.^4^ In a time when cross-national and cross-sector partnerships and collaboration were essential to stopping the spread of the disease and reducing its morbidity and mortality, fracturing and competition prevailed.

This article analyzes CEPI’s unique contribution to international collaborations across the pandemic vaccine supply chain and situates it within the much broader literature on global public health partnerships. This contribution adds to the understanding of how and when these kinds of partnerships can work for public health, especially under emergency circumstances.

In light of these movements against greater and more equal vaccine access, driven as they were by relatively narrow conceptions of national interest and profit-maximization, only a countervailing movement or movements existed driven by a more nuanced complex analysis of what would serve the community of nations and altruism or, at least, equity. These countervailing movements materialized as a partnership between international organizations that excelled in supporting national planning for vaccine rollout (the World Health Organization and UNICEF); financing vaccine procurement (Gavi, the Vaccine Alliance)^5^; and supporting COVID-19 vaccine candidates (the Coalition for Epidemic Preparedness Innovations (CEPI)). Since 2017 CEPI, the newest in this group, had built a portfolio of rights to direct use of certain vaccine platforms and products and from early 2020, it moved to leverage this portfolio specifically for COVID-19 vaccine candidates.

Together, these organizations formed the COVAX Facility, a vaccine procurement and distribution partnership. The strategy of the COVAX Facility was to join wealthy governments and their resources who would be interested in access to CEPI’s diverse vaccine portfolio with less wealthy governments who would promise certain terms in exchange for subsidized and shared access to the same portfolio. This article analyzes the role of CEPI in facilitating global access to COVID-19 vaccines. After outlining the methods that will be employed in undertaking that analysis, we conclude that global public health partnerships are critical to global health and make observations on some of the resulting implications.

The investments by COVAX’s “self-financing” governments were meant to support the procurement of vaccines by the so-called “AMC-92” governments — essentially low- and middle-income countries (“LMICs”) who could not participate in as resource-intensive ways as self-financing governments, but which nevertheless were obligated to make up-front financial commitments for purchase, to promise to indemnify companies for losses attributable to the vaccines received, and to develop a national preparedness plan for distribution.

Initially aiming at distributing 2 billion doses by the end of 2021, the COVAX effort is now largely seen as having failed to realize much of its initial promise (it had shipped approximately 1.1 billion doses by January 15, 2022, and 1.72 billion by September 15, 2022).^6^ This is in some measure because the original bargain at the heart of COVAX was undermined by the same governments who endeavored to dominate access to vaccines in the early months of the pandemic. Even with its significant portfolio of This complete sentence would read as:

Even with its significant portfolio of rights to direct use, CEPI experienced significant limitations as to the leverage they could exercise against national governments and as to both the financial incentives and coercive pressure they exerted on vaccine manufacturers. CEPI experienced significant limitations as to the leverage they could exercise against national governments and as to both the financial incentives and coercive pressure they exerted on vaccine manufacturers.

While there were many structural weaknesses with COVAX from its outset, such as the small number of wealthy governments that agreed to actually procure through the Facility, the actions and behaviour of self-financing governments and companies played a significant role in its failure to realize its potential.^7^ Going directly to companies instead of spreading investment and risk through COVAX, wealthy governments re-directed billions of doses to their populations with nearly 85 percent of the vaccine doses administered going to high-income and upper middle-income countries.^8^

Despite these challenges, CEPI made noteworthy contributions to global equitable access to COVID-19 vaccines. C. First, the relatively spontaneous and effective cooperation between major international organizations shortly after the pandemic declaration played a significant role in reducing to a material extent COVID-19’s burden of disease and death.^9^ Indeed, the Oxford/AstraZeneca vaccine, the primary COVAX export, saved 6 million lives in its first year alone.^10^ Second, future pandemic preparedness, prevention, and response will require that collaborations of this kind be sustained and effective going forward.

The purpose of this Article is to highlight and evaluate the role of CEPI and its contribution to global equitable access to COVID-19 vaccines through its established partnerships for vaccine development.

This article analyzes CEPI’s unique contribution to international collaborations across the pandemic vaccine supply chain and situates it within the much broader literature on global public health partnerships. This contribution adds to the understanding of how and when these kinds of partnerships can work for public health, especially under emergency circumstances. Section II situates this analysis in the literature on global public health partnerships. Section III describes the methodology adopted for the external review, which includes literature reviews, document reviews and interviews with key stakeholders. Section IV describes the key findings from our review in terms of both CEPI’s horizontal as well as vertical collaborations. Section V provides a brief conclusion.

## II. Global Health Partnerships

Well before COVID-19, the field and literature devoted to public-private partnerships for global health was burgeoning. Stretched funding, increasing exposure to health risks that required collective action, rapid advances in emerging technologies, and the public health repercussions of climate change laid bare the inability of one, two, or few governments to solve problems on their own.^11^ The COVID-19 pandemic underscored opportunities for public-private partnerships to enhance global health security, strengthen health systems, and address inequities to help achieve the global health targets of the Sustainable Development Goals.^12^

Despite these broad acknowledgments, the quantification and evaluation of public-private partnerships for global health as a unitary phenomenon is difficult because there is no uniformity or agreement as to their definition, qualifying criteria, or mission scope. The secondary literature is overwhelmingly case study-based. Plamondon, Brisbois, Dubent and Larson, for example, write that partnerships “involve two or more organizations collaborating together toward a common goal,” before analyzing 30 peer-reviewed articles covering public-private partnerships for global health formed with at least one partner from the Global North and one from the Global South.^13^ The consultancy Deloitte notes that “robust international partnerships to build pandemic preparedness are coalescing and becoming stronger; more governments are working with the United Nations, World Bank, WHO, and other organizations on the issue” without quantifying those partnerships nor defining them.^14^

In 2010, the WHO identified certain criteria for evaluating the requests it receives for participating in its own partnerships. Some of these criteria include “a clear added value for public health,” “appropriate and adequate stakeholder participation,” “public health goals to precede special interests of participants” and “an independent external evaluation and/or self-monitoring mechanism.”^15^ Such factors can be relevant when considering the viability and potential of a global health collaboration, but there is little evidence that they have influenced the academic study of global public health partnerships nor even, in a measurable way, WHO’s own approach. There is a vague notion that public-private partnerships for global health should incorporate best practices and principles for addressing health inequalities and inequities and promoting and actively acknowledging the limited research capacities of LMICs, but even that aim is not clearly articulated or pursued in well-known partnerships, let alone as an explicit standard expectation.^16^

Despite these limitations and ambiguities in their formal study, there is little doubt that public-private partnerships for global health are critical to helping solve pressing problems and challenges, not the least of which is prevention, preparedness, and response with respect to future pandemics.^17^ The COVID-19 pandemic transformed the global health architecture of the public and private sectors. The pandemic created novel institutional spaces for more inclusive public-private partnerships for global health, including innovations in governance, shared decision-making, risk sharing, knowledge generation, and resource planning. There have been national and global collaborations in all areas of the emergency response during the pandemic, including collaborations focused on surveillance and monitoring, public health prevention and mitigation measures, diagnostics and therapeutics, providing personal protective equipment, and increasing testing and laboratory capacities.^18^ This is also, and perhaps specially, true for COVID-19 vaccines where extensive mobilization of R&D, large-scale funding, and improved distribution networks have been enabled by collaborations such as COVAX.^19^

Formed after the West Africa Ebola public health emergency, CEPI, nominally a Norwegian non-profit association but functioning as a much more significant international organization, was established in 2017 to support the research and development of the next generation of vaccines to address WHO Blueprint diseases — those prioritized for research and development because they pose the greatest public health risk due to their epidemic potential and/or the lack or insufficiency of countermeasures.^20^ By 2019, CEPI had secured approximately USD$750 million to invest in vaccine candidates and rapid response platforms. The CEPI Equitable Access Policy recognizes that equitable access principles must be implemented throughout all stages of vaccine development, manufacture, and deployment. non-profit.^21^

While focusing in its first three years on WHO Blueprint diseases for which a commercial market was unlikely to develop, CEPI’s investments in the vaccine research and development before COVID-19 were critical to LMIC’s access to COVID-19 vaccines over the course of the pandemic. The novel partnerships it formed, and innovative commitment and incentive mechanisms it deployed, leveraged benefits for horizontal partners like Gavi and WHO, while going a significant distance toward orienting for-profit actors to shape their objectives in the direction of orienting price structures toward a public good basis.

## III. Methodology

This Article is based on a mixed methodology, including secondary literature, primary sources including CEPI’s own reports and publications, documents filed with the U.S. Securities and Exchange Commission (SEC), non-public documents made available by CEPI, and interviews with key CEPI decision-makers. The review and interviews were conducted in December 2021 as part of an external review of the extent to which equitable access has been achieved through CEPI’s COVID-19 vaccine development agreements. The resulting report is available on CEPI’s website.^22^

### A. Literature Review

#### 1. SECONDARY LITERATURE

The authors conducted a structured literature search using Bloomberg Law, Westlaw, PubMed, Excerpta Medica dataBASE (EMBASE), Cumulative index to Nursing and Allied Health Literature (CINAHL) and Global Online Access to Legal Information (GOALI) using the following predefined keywords: CEPI AND equitable access; COVAX AND CEPI; vaccine AND CEPI AND [name of partner]. From that review, the authors developed a stakeholder map for the CEPI agreements, described below. The authors also reviewed securities reports, updates, and notifications filed by partners for which such filings were required by the U.S. Securities and Exchange Commission (SEC).

#### 2. PRIMARY SOURCES

##### a. Governance and Strategy Documents

In addition to agreements and interviews facilitated by the CEPI Secretariat, the authors undertook an extensive review of CEPI’s publicly available governance and strategy documents, including the CEPI 2.0 Program Document and its annexed Results Framework and CEPI’s periodic updates to its own equitable access summary document. The authors reviewed Board meeting summaries for the period August 2016 to September 2021, the minutes from the Board’s Equitable Access Committee from November 2019 to October 2021, the Board’s Audit and Risk Committee minutes from November 2019 to March 2021, and the Board’s Executive and Investment Committee minutes from November 2019 to July 2020.

##### b. CEPI Vaccine Development, Manufacturing, Supply, and Clinical Trial Readiness Agreements

The authors were provided access to 28 agreements covering seventeen (17) CEPI partners. These agreements include Outbreak Response Funding Agreements, both Step 1 and Step 2, described below; Wave 2 Award Agreements; the Trusted Manufacturer Agreement, and various subsequent amendments to the agreements. Two sets of pre-COVID-19 agreements were also reviewed, namely the Framework Partnering Agreements (“FPA”) entered between CEPI and the University of Queensland, and between CEPI and CureVac AG.^23^

###### B. Semi-Structured Interviews

The authors conducted 10 interviews with 9 key CEPI personnel. After reviewing key personnel included in the agreements made available for review, the authors developed semi-structured interview scripts specific to the role of each CEPI Secretariat or CEPI Equitable Access Committee member. The interviews consisted of questions regarding CEPI’s Equitable Access Policy, the COVID-19 agreements entered into with partners, and the negotiations surrounding these. Interview times ranged from 30 to 90 minutes in duration. Consent was sought from the interviewees and anonymity is maintained for their responses and quotes.

The study is unique and valuable in some measure because the researchers obtained access to otherwise non-public primary sources — documents and interviews — unavailable to other researchers. However, as a result, the study is less likely to be replicated. Similarly, the study focused on those primary sources available from CEPI itself. A more comprehensive analysis of CEPI and its partnerships would involve similar access to documents and interviews with CEPI’s international organizational and commercial partners and perhaps other constituencies. These latter weaknesses represent promising areas for future research building upon this study.

## IV. Results

The following are the key findings from the review of CEPI’s COVID-19 vaccine development agreements in light of CEPI’s equitable access policy.

First, CEPI maintains a nuanced, robust commitment to equitable access, a commitment that manifested over the course of the COVID-19 pandemic, although necessarily adapted to a context in which it worked with, and alongside, international partners and commercial partners of varying size, capital, and governance structure. It did so on accelerated schedules and faced significant competition from government funders seeking or requiring bilateral arrangements. Equitable access commitments with manufacturers like Clover and Novavax differed from discrete supply input partners like adjuvant supplier Dynavax or vial producer Stevanato. Tailored to each partner and vaccine input, CEPI’s Secretariat and Equitable Access Committee deployed diverse contractual mechanisms to address their broader institutional mandate of equitable access.

Second, CEPI’s partnerships with major international organizations were shaped by agreements and memoranda of understanding concluded before the COVID-19 pandemic. These agreements facilitated the defined roles that CEPI and its partners played after the pandemic was declared. However, over the course of the pandemic, CEPI increasingly took on responsibility for facilitating discussions with regulators and negotiations with manufacturers, obligations more generally held and previously performed for main stream vaccines by its international organizational partners.

Third, with respect to its COVID-19 vaccine development, scale-up of manufacturing, and vaccine supply agreements, CEPI enjoyed the most favorable equitable access terms with newer and smaller biotechnology companies, including manufacturers, and universities, than with more established and larger ones.

Fourth, CEPI’s most visible and measurable success, other than its leadership in establishing COVAX, is its role in facilitating global access to the “Oxford/AstraZeneca” vaccine.^24^

## V. Discussion

CEPI’s global public health partnerships may be divided into “horizontal” partnerships, which we limit here to Gavi, the Vaccine Alliance, WHO and UNICEF, with whom CEPI plays a collaborative, team-based role, and “vertical” partnerships, where CEPI largely serves as a funding partner reserving certain rights that guide the partner’s conduct. CEPI maintains other partnerships which may be characterized as “horizontal” but are not covered here, like those with the African Union, the Brighton Collaboration, and the Global Health Innovative Technology Fund.^25^ For the most part, CEPI’s vertical partnerships are with for-profit companies of varying size, governance, and capital structure.

### A. Horizontal Partnerships

#### 1. WORLD HEALTH ORGANIZATION

The World Health Organization, as the preeminent international organization committed to achieving the highest attainable standard of mental and physical health for the world’s people, played a significant role in CEPI’s activities from its formation. While the conceptual inception date for CEPI varies according to historical sources, there is a consensus that an idea that had long circulated gained real momentum at a meeting hosted by the WHO in Oslo in October 2015, as the West Africa Ebola public health emergency was ebbing.^26^ WHO has always been represented in the governance structure of CEPI, and its scientific and technical bodies inform the priority-setting at CEPI, for example, establishing the list of Blueprint diseases.^27^ In CEPI’s early days, WHO wished to preserve its authority as a global norm-setter and avoid overlap with CEPI on setting scientific priorities and coordinating with regulators.

In 2017, WHO and CEPI signed a Memorandum of Understanding^28^ identifying key avenues for future collaborations, including (1) acceleration of R&D for pathogens with epidemic potential, (2) strengthening of global regulatory capacity to address outbreaks of epidemic potential and public health emergencies, and (3) emergency operations in response to a declared Public Health Emergency of International Concern (PHEIC) or where outbreaks of known/unknown pathogens are deemed to have the potential to trigger a PHEIC. Both WHO and CEPI also agreed to promote the fact that any product resulting from such collaborations would be made publicly available on reasonable terms, particularly to “the public sector of developing countries on preferential terms.”^29^

These pathways, in significant measure, laid the groundwork for cooperation during the early days of the COVID-19 pandemic. In the lead-up to the formation of the COVAX Facility, WHO provided guidance on policies, R&D technical coordination, principles of country allocation of the vaccine, and country preparedness. WHO also prepared a target product profile for COVID-19 vaccines and developed a no-fault compensation scheme as part of the time-limited indemnification and liability commitments.^30^ WHO’s Strategic Advisory Group of Experts (SAGE) on Immunization developed evidence-based immunization policy recommendations.^31^ Its Emergency Use Listing (EUL)/prequalification programmes ensured harmonized review and authorization across member states.^32^ Together with UNICEF, WHO also led COVAX’s Country Readiness and Delivery workstream which provided support to countries as they prepared to receive and administer vaccines.^33^

### 2. GAVI, THE VACCINE ALLIANCE

Primarily designed to finish the work of the WHO’s Expanded Programme on Immunisation, Gavi has advanced the coverage of routine recommended immunizations for children since its establishment in 2000,^34^ and done so through innovative funding mechanisms that gave it significant resources to play a major role in the COVID-19 response generally, and the COVAX Facility specifically. Its role has always been focused on financial support for governments, rather than individuals, research institutes, or companies. Although not originally designed as a public health emergency response organization, it has gradually become so, especially after the 2009 H1N1 and 2014-15 West Africa Ebola public health emergencies.

As with WHO, Gavi aimed to preserve its boundaries of authority and activity even while supporting CEPI’s establishment and mission. For example, procurement agreements were viewed as core to Gavi’s mission, with UNICEF serving generally as the procurement agent. CEPI’s role was, at least initially, envisioned to be stimulating, financing, and co-ordinating the development of vaccines, and enabling equitable access against potentially epidemic infectious diseases for which the market potential is limited. The financing, procurement, and regulatory aspects of Gavi’s work were to remain relatively distinct and continue within its purview. Even though CEPI’s agreements included “a right of first refusal” should one of the vaccines it supported be necessary to address an epidemic, Gavi, UNICEF or perhaps another traditional organization was to manage the beneficial outcome should that right be exercised.

Indeed, within COVAX, Gavi leads the vaccine procurement and delivery at scale workstream as well as the COVAX Advance Market Commitment, a financing mechanism to ensure access to COVID-19 vaccines.^35^ While the COVAX Facility itself has no legal personality — it cannot enter into contracts, and it is not susceptible to legal processes in any of the jurisdictions where its stakeholder organizations reside — Gavi administers the “Office of the COVAX Facility.” Gavi negotiates agreements with self-financing countries, tri-partite agreements with multilateral development banks, agreements with manufacturers with volume guarantees, assembles groups covered by its Stakeholder Agreement (and others), and performs other administrative functions.^36^

Yet over the course of the pandemic, CEPI has played roles in accelerating export permits/custom clearance, facilitating regulatory approvals, developing manufacturing workforce,^37^ and stimulating public-sector and private-sector investments in vaccine manufacturing innovations that could accelerate pandemic response and production of routine vaccines.^38^

### 3. UNICEF

The world’s largest provider of vaccines, UNICEF, is a co-leading partner in COVAX and has been the key transport delivery partner for COVID-19 vaccines for COVAX since February 2021.^39^ UNICEF works with manufacturers and partners on the procurement of COVID-19 vaccine doses, as well as on freight, logistics and storage. UNICEF also procures and transports immunization supplies such as syringes, safety boxes for their disposal, and cold chain equipment such as vaccine refrigerators and freezers. A COVID-19 Solidarity Response Fund was set up by WHO and UNICEF in 2020, and part of the resources were directed towards the efforts of CEPI on an as-needed basis.^40^

In early 2022, recognizing the urgency of turning vaccine doses into vaccinated protected communities, the WHO, UNICEF, and Gavi launched the COVID-19 Vaccine Delivery Partnership (CoVDP).^41^ The CoVDP focused foremost on the 34 countries that were at or below 10 percent coverage in January 2022, and offered urgent operational funding, technical assistance and political engagement to rapidly scale up vaccination and monitor towards targets.

For a relatively new international organization that operated with significantly fewer resources than its horizontal partners, CEPI rapidly rose to serve as an equal partner with WHO, UNICEF, and Gavi as COVAX was formed. It successfully leveraged its investments in vaccine candidates and rapid response platforms to work within WHO’s and UNICEF’s expertise as an evaluator of national readiness and Gavi’s expertise in vaccine procurement finance. CEPI contributed its significant portfolio and avoided duplication of roles, while also supplementing its partners’ responsibilities with, for example, regulatory capacity building and pricing knowledge. It is this role — as builder and investor in promising vaccine candidates — that adds significant value to the global health organizational architecture that existed before COVID-19.

### B. Vertical Partnerships

Equitable access to epidemic vaccines in the context of an outbreak has been defined by CEPI as ensuring that appropriate *“vaccines are first available to populations when and where they are needed to end an outbreak or curtail an epidemic, regardless of ability to pay.”* CEPI’s Equitable Access Policy seeks to facilitate equitable access to epidemic vaccines in three fundamental ways:

1. Funding the accelerated development and timely availability of vaccines (and including maintaining investigational stockpiles, to be used free of charge in clinical studies) where it is needed most when an outbreak occurs;
2. Coordinating with others in the global health community to enable licensure of vaccines funded by CEPI, including by securing resources for pivotal clinical trials to generate critical data and;
3. Collaborating with others in the global health community to enable the procurement, allocation, deployment and administration of licensed vaccines to protect global health, at a price that does not limit equitable access and is sustainable to the manufacturer.

The CEPI Equitable Access Policy recognizes that equitable access principles must be implemented throughout all stages of vaccine development, manufacture, and deployment.

Each of the COVID-19 vaccine agreements entered into by CEPI seeks to accomplish one or more of the following major objectives: (1) preclinical and clinical development and testing of vaccine candidates; (2) development and validation of a manufacturing process capable of producing large quantities of vaccines; (3) the supply of vaccines by that manufacturing process; and (4) supporting these aspects of development both through specific supply chain elements, like adjuvants.^42^

CEPI’s impact on rapid response was significant. The genetic sequence for COVID-19 was published on January 11, 2020. By January 23, CEPI had initiated its first three programs to accelerate the development of vaccines against the novel pathogen, when just 581 cases of the virus had been confirmed worldwide.^43^ In the space of five months, CEPI had built the world’s largest portfolio of COVID-19 vaccine candidates.

While CEPI has reached vaccine development agreements with some large companies, like AstraZeneca, for the most part its vaccine development support and partnerships have been with university research centers and small biotechnology companies. Secondary analyses suggest that this is because larger potential partners like Pfizer, Merck, and Sanofi viewed bilateral opportunities with wealthy national governments as more promising from the perspective of investor return and had fewer conditions attached.^44^ For future pandemics, the reality of large, publicly traded manufacturers, their incentives, and the broader context of wealthy governments’ seeking bilateral deals should shape how CEPI may affect planning going forward.

By now CEPI has entered into perhaps hundreds of agreements with various parties, especially if agreement amendments are included. Our analysis of CEPI’s vertical partnerships is limited therefore to those relationships CEPI lists in its own catalogue of vaccine development agreements as of March 2022.

Rather than taking a contractual, transactional approach focusing on final products, CEPI invested in, facilitated, and built end-to-end partnerships between public and private sector actors that stretched from clinical trials, to drug substance manufacturing, to adjuvants, to the vials in which the vaccines are shipped, to planning in the context of pre-license vaccine candidates. As a result of this comprehensive strategy, CEPI was an essential supporter of the three now prevalent COVID-19 vaccine platforms: mRNA, adenovirus vector-based, and protein-based vaccines.^45^

### C. Vaccine Developers

CEPI divided its initial approach to COVID-19 vaccine development agreements into two parts: Step 1 and Step 2 agreements. Step 1 agreements focused on providing “time of the essence” support to promising vaccine candidates, including scale-up of manufacturing, with broad expectations of equitable access provisions being included should the vaccine candidate proceed to Step 2. Step 2 agreements involved significantly larger investments by CEPI and correspondingly more robust commitment assurance mechanisms. These included joint monitoring committees (JMAGs) for both product development and equitable access factors; stage gate benchmarks for further support (i.e., a partner must reach pre-determined measures of progress to receive next payments); a public health license that allowed CEPI march in rights to assign project deliverables to another party should the initial partner fail to live up to its commitments; and robust dispute resolution mechanisms.^46^

This broad-spectrum approach to the vaccine development and supply chain allowed CEPI to rapidly adapt its pre-pandemic agreements to facilitate partnerships toward the end of equitable access to COVID-19 vaccines. To enable affordable and sustainable pricing, over the course of the COVID-19 pandemic, CEPI adopted several approaches successfully used in its pre-COVID-19 pandemic core portfolio and applied them to its COVAX-directed relationships with international public- and private-sector partners, primarily Gavi, which oversaw governments’ procurement, and WHO, which provided assistance in planning vaccine rollout.

CEPI tailored its framework partnering agreements (which predated the pandemic), Step 1 and Step 2 COVID-19 vaccine development agreements, manufacturing supply and reserve agreements, and clinical trial readiness agreements to the partner and public sector goal. For example, Step 1 agreements governed CEPI’s rapid support funding for promising vaccine candidates, including those of Moderna, AstraZeneca and Novavax, so that critical vaccines could progress in the development process. Step 2 agreements formalized equitable access commitments and put in place strong enforcement mechanisms to ensure that public sector investments resulted in corresponding benefits to public sector constituencies. The overarching principle in the CEPI partnering agreements was that all manufacturing output corresponding to the CEPI-funded part of development was to be offered first to the COVAX Facility. This right of first refusal of CEPI-funded vaccines ensured that the vaccine developer committed to allowing COVAX a “first-choice” in procuring vaccines.

This broad-spectrum approach to the vaccine development and supply chain allowed CEPI to rapidly adapt its pre-pandemic agreements to facilitate partnerships toward the end of equitable access to COVID-19 vaccines. To enable affordable and sustainable pricing, over the course of the COVID-19 pandemic, CEPI adopted several approaches successfully used in its pre-COVID-19 pandemic core portfolio and applied them to its COVAX-directed relationships with international public- and private-sector partners, primarily Gavi, which oversaw governments’ procurement, and WHO, which provided assistance in planning vaccine rollout. These approaches included:

- tiered pricing: paying lower prices for vaccines destined for low-income countries than prices for vaccines destined for middle- or high-income countries;
- cost-of-goods plus pricing (COGs + x%): calculating total costs for vaccine inputs and production, then applying a markup percentage to those costs to reach an equitable price;
- claims on real-time production;
- commercial benefits: a percentage of revenues realized by a partner or an agreed upon substitute for such benefits;
- “step-in rights”: the right to intervene and direct technologies or other contractual assets to a third party for satisfaction of contractual obligations.

Agreements, such as with biopharmaceutical companies CureVac and Dynavax, required the prices to be tiered based on country economic level, with supply available to all levels. CureVac was not to charge higher than the lowest price charged by it for the sale of the vaccine to a third party of a similar volume and to a country of a similar income level. In agreements with biotechnology companies Gritstone Bio Inc., Shanghai Zerun Biotechnology Co., Ltd., and SK Bioscience, the price of the vaccine was to be determined in negotiation between the vaccine developer and Gavi on behalf of the COVAX facility, based on a Cost of Good + % approach.

Even under emergency circumstances, CEPI deployed a diverse set of mechanisms to address equitable access. These included its Joint Monitoring and Evaluation Committee (JMAG), repayment requirements under specified circumstances, and robust, real-time information sharing commitments.^47^ CEPI’s partnership with the University of Oxford and AstraZeneca on COVID-19 leveraged CEPI’s prior investments in Oxford’s ChAdOx1 vaccine platform, which had been applied to a number of diseases, including MERS, Lassa and Nipah. These investments in the work on MERS in particular enabled Oxford to pivot quickly to work on COVID-19. According to analyses in *Nature* and the *Economist,* Oxford’s ChAdOx1 vaccine, which enjoyed early and substantial support from CEPI, is not only the most widely available and administered, but it has also saved more lives than any other.^48^ As of January 21, 2022, 2.5 billion doses of the vaccine have been supplied to 170 countries across the world, with approximately two-thirds of these to low-and lower-middle-income countries.^49^ Oxford’s agreement with AstraZeneca, shaped by Oxford’s agreement with CEPI, included the company’s commitment to prioritize LMICs and to charge a non-profit price during the pandemic.^50^ CEPI’s own agreement with AstraZeneca gave it independent authority to audit the no-profit calculation undertaken by AstraZeneca.

CEPI was also one of the first investors in Novavax’s COVID-19 vaccine candidate, NVX-CoV2373, and has provided up to US $399 million in funding to accelerate development and manufacturing of the vaccine for equitable global allocation.^51^ In December 2021, Novavax’s vaccine became the world’s first protein-based COVID-19 vaccine to receive Emergency Use Listing and on July 13, 2022 it was granted emergency use authorization by the U.S. FDA.

CEPI’s long list of collaborations with COVID-19 vaccine developers includes Moderna, in which it made an investment of nearly one million USD in January 2020, Inovio Pharmaceuticals, Inc, University of Hong Kong, The Institut Pasteur, and Clover BioPharma.^52^ CEPI’s support for Clover’s COVID-19 vaccine candidate also involved negotiation not only with companies, but with national and sub-national governments. Notably CEPI facilitated support from partners from across the globe including Australia, Italy, Netherlands, and the United States of America.

#### D. Manufacturing Capacity

Vaccine development, production and delivery is an expensive, high-risk, complicated, and time-consuming process. Typically, manufacturers do not invest in scaling up manufacturing capacity unless efficacy is proven, and the vaccine is licensed for commercial sale. Given the urgency and unique circumstances of the COVID-19 pandemic, the world could not have waited until the efficacy of vaccine candidates were proven before investing in scaling out production capacity without unacceptably massive loss of life. CEPI collected public-sector contributions and made “at-risk” investments in technology transfer to increase available manufacturing capacity prior to the results of efficacy trials.^53^

CEPI not only supported Novavax’s vaccine candidate development, but also partnered with SK Bioscience to reserve manufacturing capacity for the Novavax (and other) vaccines, support SK Bio’s own recombinant protein vaccine candidate against new virus variants, and planned expanded manufacturing capacity with sub-national governments in Korea.^54^

In December 2020, CEPI and Biological E Limited announced a collaboration to advance the development and manufacture of Bio E’s COVID-19 subunit vaccine candidate.^55^ Both agreed that vaccine output funded by CEPI’s investment was to be made available for procurement and allocation, if proven to be safe and effective, through COVAX.

To advance the development and delivery of vaccines manufacturing in Africa, in January 2022, CEPI agreed to provide strategic and technical support to Institut Pasteur de Dakar’s (IPD) MADIBA project, a regional manufacturing hub for COVID-19 and other vaccines in Dakar, Sénégal.^56^ CEPI will also advise on the implementation of an innovative vaccine filling and delivery solution, licensed from MedInstill/INTACT Solutions and developed with CEPI’s funding and support.

#### E. Adjuvants and Glass Vials

CEPI and Dynavax Technologies Corp. entered into a partnership on 29 January 2021 to supply its proprietary CpG 1018 adjuvant to CEPI Partners.^57^ To advance the at-risk manufacture of the vaccine, CEPI provided an interest-free, forgivable and unsecured loan, recoverable upon product sales. The forgivable loan structure and time horizon for use of CpG1018 appeared appropriate safeguards for CEPI’s interest in equitable access to support CEPI’s other supported vaccine candidates.

CEPI’s holistic approach with collaborations across key actors have helped vaccine manufacturers speed up vaccine development. For example, CEPI also provided Clover with supplies including adjuvant from Dynavax (USA), and vials from Stevenato Group (Italy). In June 2020, CEPI signed an agreement with Stevanato Group for the supply of 100 million Type 1 Borosilicate glass vials to hold up to 2 billion doses of a vaccine against the COVID-19 virus.^58^ The high-quality glass vials that CEPI secured from Stevanato Group were to store 20 doses per vial (2 billion doses in total). In addition to manufacturing inputs, CEPI supported next steps in the regulatory process, especially clinical trial preparedness.

#### F. Clinical Trials

As the pandemic progressed, CEPI also supported new or variant-specific technologies, booster doses trials and trials of “mix-and-match” combinations of COVID-19 vaccines to aid in the response. In one such program in December 2021 with SK Bioscience, CEPI agreed to provide financial support for the development of a vaccine candidate based on SK’s nanoparticle vaccine platform to elicit immune responses that could protect against multiple variants, including SARS-CoV & SARS-CoV-2.^59^ The funding supported design, preclinical studies, Phase I/II clinical trials, production of necessary clinical trial material, and process and analytical development. This type of agreement represents a more complex integration of CEPI support across the vaccine development process including, for example, adjuvant supply, vaccine development, and scale-up of manufacturing.

In November 2021, CEPI and the Consortium led by Aga Khan University announced a new collaboration to conduct a clinical trial of heterologous COVID-19 vaccines in Pakistan.^60^ The clinical trial will assess the safety and immunogenicity of mix-and-match combinations of three vaccines that are deployed in Pakistan, developed by AstraZeneca, Sinopharm, and CanSinoBIO. Similarly, CEPI announced in April 2022 an agreement to co-fund the ongoing global pivotal Phase 3 clinical trial of Vaxxinity, Inc.’s next generation UB-612 COVID-19 vaccine candidate as a heterologous booster dose.^61^

CEPI awarded $6.3 million to the Sabin Vaccine Institute in May 2022 for a clinical trial to evaluate the immunogenicity and safety of lower, fractional doses of registered COVID-19 vaccines used as a booster dose.^62^ Grant management support will be provided by PATH, an international nonprofit global health organization.

More recently, in June 2022, CEPI and the Oxford Vaccine Group (OVG) in the UK and Brazil partnered to conduct a clinical trial in Brazil to investigate the impact of administering reduced COVID-19 booster shots instead of full doses.^63^ The vaccines involved in this study are being distributed through COVAX, primarily in LMICs. In line with CEPI’s Equitable Access Policy, all data from the clinical trial will be shared through open-access publications and via scientific meetings.

CEPI’s partnerships with vertical partners were fundamentally shaped by the behavior of high-income governments and the publicly traded firms, mostly located in the latter’s sovereign territory. Given the limitations that these constituencies imposed — including export controls but primarily the preference of wealthy governments to pursue bilateral deals rather than contribute to COVAX — CEPI should use its relatively positive outcomes earned from university and smaller biotechnology companies as part of its planning going forward. CEPI may, for example, identify inputs and contractual arrangements that are more resistant to government threats, or enter into three-part agreements with developers and governments that clarify its rights.

## VI. Conclusion

Despite the near-miraculous timeframe within which safe and efficacious vaccines were developed and authorized for emergency use, by the end of 2021 more than 95 percent of the global population lacked access to the first dose of life-saving COVID-19 vaccines, even as governments in wealthy countries were recommending and mandating booster vaccines for those already inoculated. Before and during the COVID-19 pandemic, CEPI built both horizontal and vertical partnerships that allowed it to leverage its legal rights and its global presence toward making COVID-19 vaccines available to populations worldwide consistently with its equitable access mission. While this was primarily oriented toward procurement and distribution through COVAX, CEPI also used its partnerships to orient global supply chains and many vertical partners toward ends consistent with COVAX’s even though not technically run through it. The most important and successful of these partnerships was that with Oxford and AstraZeneca, to date the most widely distributed vaccine that has saved the most lives. More broadly, CEPI experienced significant limitations in what it was able to accomplish. Given its experience, its future agreements and partnerships should assess and navigate this geopolitical and economic terrain carefully. As CEPI embarks on its second-stage planning and its ambitious 100-days mission — in which the world would be ready to start deploying a future pandemic vaccine within 100 days — its existing horizontal and vertical partnerships will be instrumental in achieving both equitable access to those vaccines and enhanced global health security.

## Note

In December, 2021, the O’Neill Institute for National and Global Health Law housed at Georgetown University conducted an external review of how equitable access has been achieved through CEPI’s COVID-19 vaccine development agreements. The review focused on evaluating the implementation of CEPI’s Equitable Access Policy in COVID-19 vaccine agreements, the advances made towards CEPI’s commitment to enabling equitable access, and to enable open access to data, results and publications arising from its funding and facilitate access to materials to accelerate vaccine development. The authors acknowledge support by CEPI for the research summarized in this work. However, the study was undertaken independently.
